# Wafer-scale epitaxial modulation of quantum dot density

**DOI:** 10.1038/s41467-022-29116-8

**Published:** 2022-03-28

**Authors:** N. Bart, C. Dangel, P. Zajac, N. Spitzer, J. Ritzmann, M. Schmidt, H. G. Babin, R. Schott, S. R. Valentin, S. Scholz, Y. Wang, R. Uppu, D. Najer, M. C. Löbl, N. Tomm, A. Javadi, N. O. Antoniadis, L. Midolo, K. Müller, R. J. Warburton, P. Lodahl, A. D. Wieck, J. J. Finley, A. Ludwig

**Affiliations:** 1 Ruhr-Universität Bochum, Lehrstuhl für Angewandte Festkörperphysik, Universitätsstraße 150, 44801 Bochum, Germany; 2 Walter Schottky Institut and Physik Department, Technische Universität München, Am Coulombwall 4, 85748 Garching, Germany; 3 Munich Center for Quantum Science and Technology (MCQST), Schellingstr. 4, 80799 Munich, Germany; 4 Center for Hybrid Quantum Networks (Hy-Q), Niels Bohr Institute, University of Copenhagen, Blegdamsvej 17, DK-2100 Copenhagen, Denmark; 5 Department of Physics, University of Basel, Klingelbergstrasse 82, CH-4056 Basel, Switzerland; 6 Walter Schottky Institut and Department of Electrical and Computer Engineering, Technische Universität München, Am Coulombwall 4, 85748 Garching, Germany

**Keywords:** Design, synthesis and processing, Quantum dots, Quantum dots, Surface patterning, Quantum dots

## Abstract

Precise control of the properties of semiconductor quantum dots (QDs) is vital for creating novel devices for quantum photonics and advanced opto-electronics. Suitable low QD-densities for single QD devices and experiments are challenging to control during epitaxy and are typically found only in limited regions of the wafer. Here, we demonstrate how conventional molecular beam epitaxy (MBE) can be used to modulate the density of optically active QDs in one- and two- dimensional patterns, while still retaining excellent quality. We find that material thickness gradients during layer-by-layer growth result in surface roughness modulations across the whole wafer. Growth on such templates strongly influences the QD nucleation probability. We obtain density modulations between 1 and 10 QDs/µm^2^ and periods ranging from several millimeters down to at least a few hundred microns. This method is universal and expected to be applicable to a wide variety of different semiconductor material systems. We apply the method to enable growth of ultra-low noise QDs across an entire 3-inch semiconductor wafer.

## Abstract (web summary)

Nucleation control of self-assembled quantum dots is challenging. Here, the authors employ conventional molecular beam epitaxy to achieve wafer-scale density modulation of high-quality quantum dots with tunable periodicity on unpatterned substrates.

## Introduction

Spontaneous pattern formation is common in many natural systems having characteristic sizes ranging from the atomic to the cosmic scale. Typically, spontaneous ordering arises in inherently nonlinear systems due to the complex interplay of thermodynamic and dissipative processes that lead to minimization of local free energies^1^. In the context of the lattice-mismatched growth of III-V semiconductor nanostructures, this principle is exploited to create defect-free nanoscale islands of low bandgap materials surrounded by a wider bandgap matrix, called self-assembled quantum dots (QDs)^2,3^. Such nanostructures are versatile building blocks that are widely used in advanced opto-electronic device technologies, such as highly performant LEDs^4^ and energy-efficient nano-lasers^5^, as well as discrete quantum components like non-classical light sources for use in photonic quantum technologies^6–8^. Key factors for the device integration of such QDs are their size, shape and composition, and control of their areal density^9^. For example, exploiting their narrow emission linewidth for modal gain in nano-lasers requires high-density regions to provide sufficient gain^10^, whereas in quantum technology, highly-efficient single-photon sources require low density and positioning over the length scale of the optical wavelength^11,12^. Key metrics for the QD quality are near-transform limited emission and absorption linewidths and near-unity single-photon indistinguishability (see ref. ^6^ and references therein).

The mechanism that drives self-assembled QD growth is based on strain relaxation during heteroepitaxy of materials having different lattice constants. In the case of InAs on GaAs, strain builds up due to the 7% larger lattice constant of InAs compared to GaAs, inducing a change of growth mode from layer-by-layer growth (Frank-Van der Merwe) to layer-plus-island Stranski-Krastanov (SK) growth^13,14^. The exact moment of nucleation is heavily influenced by the growth conditions^15,16^. Due to a steep onset of the nucleation at a critical InAs layer thickness^14^, low QD density control is challenging.

We find that controlling the surface roughness at the atomic scale is a key factor for engineered QD nucleation that has been largely neglected until now. Compared to atomically smooth growth surfaces, rougher surfaces enhance the QD nucleation probability^17–19^. It is well known that atomically flat substrates successively undergo cycles of roughening and smoothening as the fractional completion of each monolayer changes; non-integer filling of each atomic layer results in atomically rough surfaces, that smoothen as the monolayer is completed. This property is utilized, for example, in reflection high-energy electron diffraction (RHEED) growth rate analysis, a standard method in e.g. MBE^20^.

Here, we exploit the impact of roughness on QD nucleation by growing layer thickness gradients prior to the deposition of QDs, thus creating in situ integer/non-integer layer numbers and roughness modulations. As a result, QD density modulations over the entire wafer are created. By controlling (i) the orientation of the substrate relative to the effusion cell, (ii) the deposition amount and interrupt time and (iii) the substrate temperature, we show that we can precisely engineer the roughness distribution to produce a variety of QD density patterns on the wafer in one and two dimensions.

## Results

**Fig. 1:**
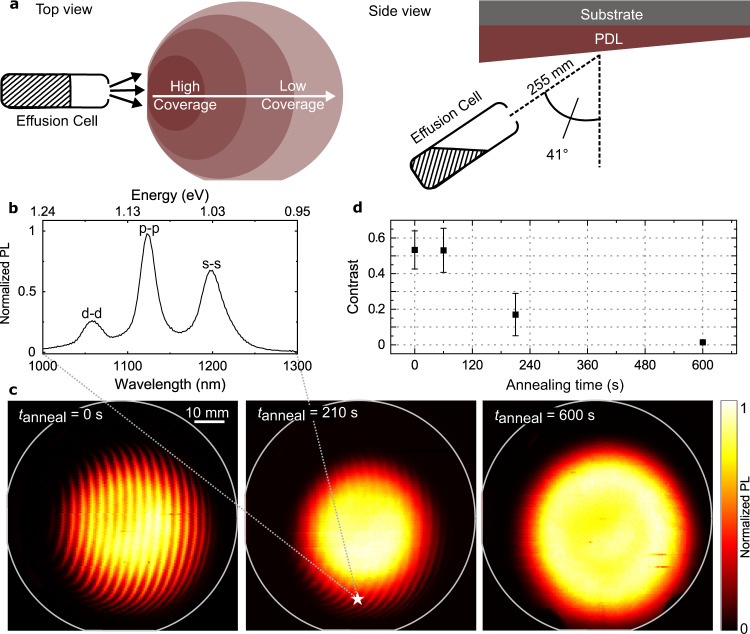
Effusion cell geometry and QD density modulation. **a** Schematic representation of the gradient of material coverage on the substrate in top view (left) and geometrical configuration of the gallium effusion cell inside the MBE growth chamber viewed from the side (right). **b** Ensemble photoluminescence (PL) spectrum at 100 K with a laser spot size of ~100 µm from a 210 s annealed sample (white star). The different peaks correspond to the different dipole and parity allowed interband transitions between orbital states. **c** False color PL maps recorded from 3″ wafers with a nominally 15 nm thick GaAs pattern defining layer (PDL). The QD PL intensity is spectrally integrated over the region between 1000 and 1300 nm. The wafers were annealed before the QD growth for 0 s, 210 s, and 600 s, respectively. **d** Michelson contrast at medium densities versus the annealing time. Error bars represent the standard deviation.

The key step in our sample preparation is the growth of a gradient layer, that is termed a pattern defining layer (PDL). This is illustrated in Fig. 1a: by depositing material from an inclined effusion cell while substrate rotation is stopped, a thickness gradient is created^21^. We grow such a PDL consisting of a GaAs gradient layer with a nominal thickness of 15 nm at the wafer center, corresponding to an overall thickness difference of 22 monolayers (ML) across the entire wafer. After deposition of the PDL, the substrate temperature was reduced from 600 °C to 525 °C, thereby (i) enabling InAs deposition without excessive desorption^22^ and (ii) preserving the surface morphology of the PDL. On top of the GaAs PDL, self-assembled QDs were grown by depositing InAs and subsequently capped with GaAs (refer to Ludwig et al.^23^ for QD growth details and method section for further preparation).

A typical ensemble photoluminescence (PL) spectrum recorded from such QDs is presented in Fig. 1b. It exhibits three distinct emission peaks corresponding to parity allowed interband transitions between the orbital states in the QDs. Figure 1c (left) shows a typical map of the PL intensity of the entire QD emission integrated within the spectral range of 1000–1300 nm at each point of a wafer. The data reveal a clear modulation of the integrated QD emission intensity along the horizontal direction, revealing a curved stripe pattern. To investigate the impact of the surface roughness on the local QD density, we performed a series of annealing tests in which the substrate and PDL was held for a time *t*_anneal_ at *T*_substrate_ = 600 °C in order to smooth it before deposition of the QD layer. Figure 1c compares data recorded for different annealing times immediately before the substrate temperature was reduced for the InAs deposition. In contrast to the clear modulation for the *t*_anneal_ = 0 s reference sample, the pattern progressively disappears after *t*_anneal_ = 210 s of annealing. For the longest investigated annealing time of *t*_anneal_ = 600 s, all intensity modulation other than the inhomogeneity due to the inherent indium cell flux distribution disappears. We observe the strongest intensity modulation in regions of just the critical amount of deposited InAs for QD nucleation and for this coverage the local QD density varies from zero to finite values.

To compare the patterns quantitatively, we calculated the Michelson contrast at comparable densities for the different samples (Supplementary Information). Plotting this contrast versus the annealing time in Fig. 1d, we observe a stable contrast for the first 60 s, after which it diminishes. The complete disappearance of the QD density modulation for an annealing time of 600 s is comparable to the coalescence time of surface islands and holes reported by Franke et al.^24^ in surface morphology studies. This observation suggests a link between the local surface roughness on the wafer and the QD density, similar to studies of growth on vicinal substrates^25,26^.

**Fig. 2:**
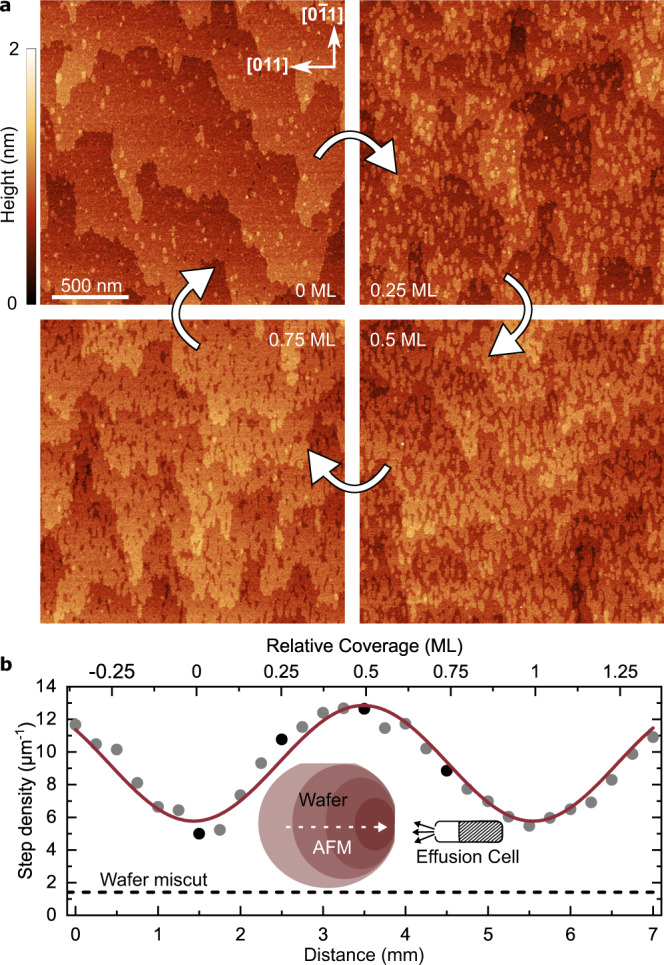
Atomic force microscopy measurements of a surface pattern defining layer. **a** AFM maps of GaAs surfaces after a coverage of 0, 0.25, 0.5 and 0.75 ML relative to the location of the lowest step density. **b** Step density (circles) determined from AFM measurements along the thickness gradient of the PDL (white dashed line in the inset wafer illustration) and sinusoidal fit (red line). The black circles correspond to the four images in (**a**). The calculated step density originating from the wafer miscut in the horizontal [011] direction is marked by the dashed black line and is determined from average terrace widths.

To confirm this expectation, we performed experiments in which the growth was stopped before deposition of the QD layer, thereby creating a GaAs PDL on the surface. Atomic force microscopy (AFM) measurements were performed on the surface at multiple points along the thickness gradient. Figure 2a shows typical data recorded at different stages of GaAs coverage relative to a smooth (integer value) surface. We define an integer number of layers by choosing an arbitrary starting point of 0 ML at the location where the lowest step density is measured and observe a clear progression of the surface in accordance with layer-by-layer growth, similar to surface studies by Bell et al.^27^. Completely finished monolayers, termed 0 ML here, show smooth surfaces covered with only a few small islands and holes. The wide monolayer terraces as seen in the first image (0 ML) occur due to the unintentional wafer miscut and consist of monolayer steps. The width of the steps is on average 500 nm, which corresponds to a 0.03° miscut. Increasing the coverage by 0.25 ML results in the formation of ~60 nm wide islands which are elongated along the $[0\bar{1}1]$ direction. At 0.5 ML coverage, these islands merge which makes the wafer miscut only barely visible. This finding indicates that step flow growth, meaning the growth of the miscut-related preexisting steps, is insignificant. Adatoms do not accumulate at the miscut steps but rather nucleate on top of them. Lastly, at around 0.75 ML the merged islands leave behind small, elongated gaps. The step densities along the [011] direction determined from these AFM images are presented in Fig. 2b, exhibiting a variation between 6 and 13 steps/µm. The lowest measured step density is still higher than the ~1.4 steps/µm in horizontal direction stemming from the miscut, since some finite roughness (islands or holes) always exists if the surface is not annealed. Comparing the density modulation periodicity of 3 mm observed in these AFM measurements with the periodicity of the PL intensity, it is clear that each stripe in the PL maps is the result of the variation in step density between two integer monolayers of GaAs of the underlying PDL.

To prove that the PL intensity pattern is a direct consequence of the modulated QD density and to examine the morphology of such QDs, we performed AFM measurements of another sample, where the growth was stopped immediately after the deposition of the InAs QD layer.

**Fig. 3:**
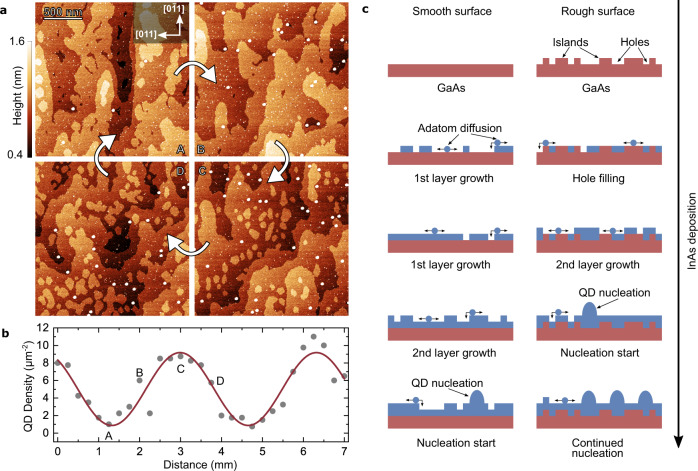
Atomic force microscopy measurements of surface QDs and enhanced nucleation schematic. **a** AFM images of surface QDs along the PDL GaAs gradient direction. **b** QD densities determined from AFM images along the PDL direction (gray dots) and sinusoidal fit (red line). The images in (**a**) are marked by the corresponding letters and represent one PDL, i.e. GaAs ML cycle. **c** Schematic illustrating InAs layer (blue) development under increasing InAs deposition on a smooth and rough GaAs surface (red). Adatom diffusion (blue dots) takes place on the surface. QD nucleation (blue domes) on rough surfaces starts earlier than on smooth surfaces.

Figure 3a shows typical AFM measurements performed on these samples along the thickness gradient in a region of low QD density. For the underlying In(Ga)As wetting layer, we observe a similar modulation of the atomic island roughness arrangement as for the GaAs PDL of the sample discussed in relation to Fig. 2. In contrast to the GaAs surface, the In(Ga)As shows much larger islands which results from the increased surface diffusion length of InAs^14^. However, the most striking observation in these data pertains to the modulation of the QD density. The deposited InAs is barely enough to induce QD nucleation (~1.6 ML), as evident from the overall low QD density and the presence of a second species of smaller QDs^28^. The density of the larger QDs, to which we attribute the observed PL signal, is plotted in Fig. 3b and is modulated between ~1 and 10 QDs/µm². We find that the QDs tend to be slightly larger in size at low-density regions (Supplementary Information). Furthermore, we do not observe a step erosion of the wetting layer as described by Placidi et al.^29^ or preferred QD nucleation at the step edges as described by Leon et al.^26^, since most QDs seem to be on terraces away from step edges. Instead, this observation suggests that the dominant process for nucleation can be traced to filling of holes in the GaAs PDL by InAs.

As illustrated schematically in Fig. 3c, in our suggested model we attribute the increased QD density to a local reduction in the effective critical InAs amount for QD nucleation. When depositing InAs on top of GaAs surfaces, layer-by-layer growth occurs on smooth surfaces, while the holes observed in the AFM images are filled by InAs and subsequently overgrown by extended monolayers of InAs. As a result of the effective increase in the local layer thickness, growth at the InAs filled locations experiences a higher strain than at the thinner layers on GaAs where holes are not present. Thus, the strain-induced QD nucleation is more likely to occur above such holes. As a consequence, hole-dominated surfaces, i.e. with GaAs surface coverage >0.5 ML, tend to show higher QD density, while smooth or island-dominated surfaces with a coverage <0.5 ML GaAs show smaller QD densities, resulting in a modulation of the QD density. The step density of the underlying GaAs surface is hidden by the In(Ga)As wetting layer. Hence, determining the precise roughness of the PDL from the wetting layer is challenging due to the difficulty of determining (i) intermixing of indium and gallium in the wetting layer, (ii) re-evaporation of In and (iii) the exact InAs amount used for QD nucleation.

**Fig. 4:**
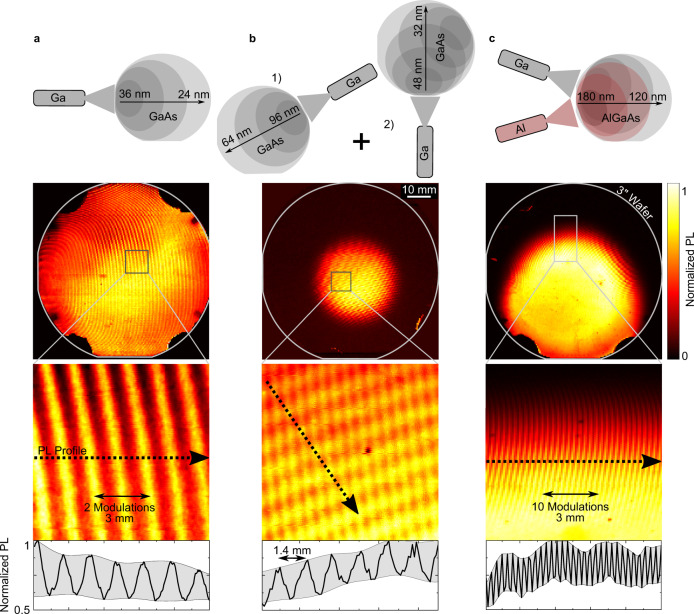
Demonstration of epitaxial pattern control. QD PL intensity maps of **a** 30 nm GaAs PDL, **b** superposition of 80 and 40 nm GaAs PDL, and **c** 150 nm AlGaAs PDL. High-resolution maps of the marked areas are shown below. The normalized PL intensity along the black dotted line of the respective zoom-ins is presented in the bottom row.

We continue to demonstrate control over the pattern formation by tailoring nominal thickness gradients along different axes of the 3-inch wafer. Figure 4a shows that doubling the PDL thickness from 15 to 30 nm halves the modulation period, from 3 mm to 1.5 mm. This provides a unique way to measure directly the PDL cell effusion profile of any MBE system with sub-monolayer precision across the entire wafer, simply by recording the spatial dependence of the QD PL intensity.

The data presented in Fig. 4b was recorded from a sample for which we first grew an 80 nm thick PDL to define a specific axis along which the density is modulated, followed by a 60 s smoothing growth interruption, before growing a second 40 nm PDL along an axis oriented at a relative angle of 120° to the first. The smoothing growth interruption between the growth of the two PDLs is necessary to provide partially smoothed areas interspersed in rough regions for the second layer modulation while still retaining some of the roughness modulation of the first layer. These observations clearly show that our methods are highly flexible, allowing the design of a specific 2D pattern across the entire wafer before QD growth.

We now continue to demonstrate that our method is equally applicable to PDLs formed in ternary alloys. Hereby, we present in Fig. 4c QD PL data recorded from a PDL defined by depositing 150 nm of Al_0.33_Ga_0.67_As and a 2.5 nm thick GaAs buffer layer before depositing the QDs. Clearly the underlying PDL roughness modulation is preserved. Similar results were obtained using a pure AlAs PDL (Supplementary Information). Furthermore, the data presented in Fig. 4c show how, by increasing the PDL thickness from 30 nm to 150 nm, the modulation period can be further reduced to 300 µm. This demonstrates that the roughness modulation is preserved at the growth surface, even after >500 MLs have been deposited. For instance, this is much more than would be observable in RHEED oscillations for the specific growth conditions used here.

The data presented in Fig. 4 clearly show that spatial roughness modulation is an exceptionally useful tool to achieve low QD densities on a full wafer. Furthermore, the approach is universal and can be used for gradient layer-by-layer growth using any binary or ternary alloy in the group-III arsenide family. Thus, we conclude that the roughness modulation method presented here should also be fully applicable to other materials systems that involve strain-driven self-assembly^13^. Beyond this, first growth trials using our MBE system hint towards ordering effects of metallic droplets on an AlGaAs PDL which could, for example, be used for droplet epitaxy or hole etching and subsequent local droplet-etched dot growth^30–32^ (Supplementary Information).

Even smaller modulation periods than those presented in Fig. 4 could be achieved by using steeper material gradients. This would require either thicker layers, shallower angles of the wafer relative to the cell, or partial flux shadowing. We anticipate that the physical limitation of our method is most likely defined by the point at which the adatom diffusion length becomes comparable to the roughness modulation period. At this point, layer-by-layer growth evolves to step-flow growth^33^, roughness-related puddles become filled, and the roughness modulation vanishes. The diffusion lengths for gallium adatoms on GaAs range between 2 nm up to 1 µm depending on growth parameters such as substrate temperature and arsenic flux^34,35^. This makes us confident that epitaxial control of QD nucleation at sub-micron length scales is reachable, possibly down to the optical wavelength in the medium.

Achieving in situ alignment on this length scale has the potential to be transformative for quantum technology applications, since QDs grown using the scheme presented here have shown near-transform limited linewidths and near-unity photon indistinguishability proving the excellent optical quality (Supplementary Information and refs. ^11,12,36–44^). We believe this low-noise environment is facilitated by the absence of prolonged growth breaks thereby preventing the incorporation of impurities and creation of crystal defects^45^ that can trap and release charge carriers.

We note that in the parameter regime of step-flow growth periodic roughness variations are absent. Thus, we conclude the PDL technique is not readily applicable there. In the standard regime of layer-by-layer growth, the roughness modulation method presented in this work demonstrates a simple and efficient way to control the density of low-noise, high-quality self-assembled QD nanostructures for advanced opto-electronic and quantum photonic applications.

## Methods

### Sample growth

All samples were grown on undoped (100) surfaces of 3″ GaAs wafers with a miscut <0.1° (as specified by the vendor) using a custom horizontal MBE System. Before growth, wafers were heated to 640 °C under an arsenic atmosphere of $9.6\times {10}^{-6}$ Torr beam equivalent pressure (BEP) to remove surface oxides. An arsenic valved cracker was employed, operating at 700 °C, providing primarily As_4_. We used growth rates of 0.2 nm/s for GaAs, 0.1 nm/s for AlAs and ~0.013 nm/s for InAs. We prepared the wafers by deposition of a buffer consisting of a 50 nm thick GaAs layer and a 30 period superlattice of 2 nm AlAs and 2 nm GaAs, followed by another 50 nm GaAs buffer layer, all grown at 600 °C and an arsenic BEP of $9.6\times {10}^{-6}$ Torr. For electrical contact, we grew a Si-doped back contact with a doping concentration of $2\times {10}^{18}{{{{{\rm{c}}}}}}{{{{{{\rm{m}}}}}}}^{-3}$, followed by a 5 min annealing break and a 5 nm GaAs layer at 575 °C to prevent silicon segregation. After an increase back to 600 °C, a pattern defining layer (PDL) of GaAs, AlAs or a ternary alloy Al_*x*_Ga_(1−*x*)_As was grown (Supplementary Information Table 1). To avoid direct QD growth on ternary alloys, a 2.5 nm thick spacer layer of GaAs was deposited on the Al-containing PDLs. After the PDL deposition, the substrate temperature was reduced from 600 °C to 525 °C by 50 °C in 30 s, another 25 °C in 60 s and followed by a 60 s settling break. Quantum dots were grown in SK-growth mode at 525 °C substrate temperature and an arsenic BEP of $6.8\times {10}^{-6}$ Torr. For this, InAs was deposited in cycles of 4 s growth, followed by a 4 s break, amounting to a total of 12 cycles and resulting in coverages of 1.6–1.8 ML. During the first 2–4 cycles, the wafer rotation was stopped so that the indium effusion cell was oriented towards the wafer big flat. After an additional 20 s break, the QDs were capped with a 10 nm thick layer of GaAs, 130 nm AlGaAs and 5 nm GaAs. QDs in samples where the dot height was reduced to 3 nm due to the indium flushing method have an emission wavelength of 910 to 960 nm. For this, the QDs were capped with 3 nm GaAs, after which the substrate temperature was linearly increased to 600 °C in 60 s, while the arsenic BEP was linearly increased to $9.6\times {10}^{-6}$ Torr. For more in depth sample and QD growth details, the reader is referred to Ludwig et al.^23^.

### Photoluminescence measurements

Photoluminescence (PL) measurements were performed by exciting samples with a 518 nm laser with a spot size of ~100 µm in diameter with total excitation powers between 1 and 20 mW. Liquid nitrogen was used to cool a 3″ cold-finger inside a cryostat which is fixed to two stepping motors for position control. Thus, the sample temperature for all PL measurements was approximately 100 K. A spectrometer equipped with a Si-CCD was used for the measurement of wavelengths between 340 and 1020 nm, combined with an InGaAs line array detector for 900 to 1715 nm.

### Atomic force microscopy

For atomic force microscopy (AFM) measurements, a Bruker Dimension Icon system was used in PeakForce tapping mode. Areas of 2×2 µm² with a resolution of 512 × 512 px^2^ were scanned.

The step density was extracted by counting how many times the derivative along the [011] direction surpasses a set threshold for each measured line during AFM.

### Reporting summary

Further information on research design is available in the Nature Research Reporting Summary linked to this article.

## Supplementary information

Supplementary Information

Description of Additional Supplementary Files

Reporting Summary

## Data Availability

The data that supports this work is available from the corresponding author upon reasonable request.

## Code availability

The code that has been used for this work is available from the corresponding author upon reasonable request.
