# Supplementary material for: Wafer-scale epitaxial modulation of quantum dot density
Source: Nat Commun. 2022 Mar 28;13:1633. doi: 10.1038/s41467-022-29116-8 (PMC8960873; doi:10.1038/s41467-022-29116-8)
Supplement: Supplementary file 1 — Supplementary Information [file 41467_2022_29116_MOESM1_ESM.pdf]

Supplementary Information to:

Wafer-Scale Epitaxial Modulation of Quantum Dot Density

N. Bart[1]*, C. Dangel[2,3]*, P. Zajac[1], N. Spitzer[1], J. Ritzmann[1], M. Schmidt[1], H. G. Babin[1], R. Schott[1], S. R. Valentin[1], S. Scholz[1], Y. Wang[4], R. Uppu[4], D. Najer[5], M. C. Löbl[5], N. Tomm[5], A. Javadi[5], N. O. Antoniadis[5], L. Midolo[4], K. Müller[3,6], R. J. Warburton[5], P. Lodahl[4], A. D. Wieck[1], J.J. Finley[2,3], and A. Ludwig[1†]

1- Ruhr-Universität Bochum, Lehrstuhl für Angewandte Festkörperphysik, Universitätsstraße 150, 44801 Bochum, Germany

2 – Walter Schottky Institut and Physik Department, Technische Universität München, Am Coulombwall 4, 85748 Garching, Germany

3 - Munich Center for Quantum Science and Technology (MCQST), Schellingstr. 4, 80799 Munich, Germany

4 - Center for Hybrid Quantum Networks (Hy-Q), Niels Bohr Institute, University of Copenhagen, Blegdamsvej 17, DK-2100 Copenhagen, Denmark

5 - Department of Physics, University of Basel, Klingelbergstrasse 82, CH-4056 Basel, Switzerland

6 - Walter Schottky Institut and Department of Electrical and Computer Engineering, Technische Universität München, Am Coulombwall 4, 85748 Garching, Germany

*These authors contributed equally to this work.

[†]Correspondence to: Arne.Ludwig@rub.de

Supplementary Table 1 Overview of sample growth parameters. PDL is the pattern defining layer.

Figure	PDL	PDL Temperature (°C)	Smoothing break (s)	Wafer size (")	Flushing (nm)	Internal #
1c (left), S3	15 nm GaAs	600	0	3	-	15154
1c (middle)	15 nm GaAs	600	210	3	-	15167
1c (right)	15 nm GaAs	600	600	3	-	15155
2	15 nm GaAs	600	0	3	-	15435
3, S10	15 nm GaAs	600	0	3	-	15424
4a	30 nm GaAs	600	0	3	3	15258
4b	1) 80 nm GaAs, 2) 40 nm GaAs	600	1) 60, 2) 0	3	-	15189
4c	150 nm Al33Ga67As + 2.5 nm GaAs	600	0	3	3	15095
S1a, S2	15 nm GaAs	525	30	3	3	15097
S1b	75 nm Al33Ga67As + 2.5 nm	600	0	3	3	15074
S1c	15 nm AlAs +2.5 nm GaAs	600	0	3	3	15088
S4, S5	15 nm GaAs	600	0	3	-	15288
S6	30 nm AlGaAs	630	30 + 120	3	-	15182
S7 and Refs [3,4]	31 nm GaAs	600	0	3	2.4	14843
S8 and Refs [5-9]	35 nm GaAs + 10 nm AlGaAs	600	30	3	2.8	14813
S9 and Refs [10-13]	40 nm GaAs	600	30	3	2.2	15027

Additional samples with different Al-concentration and geometry

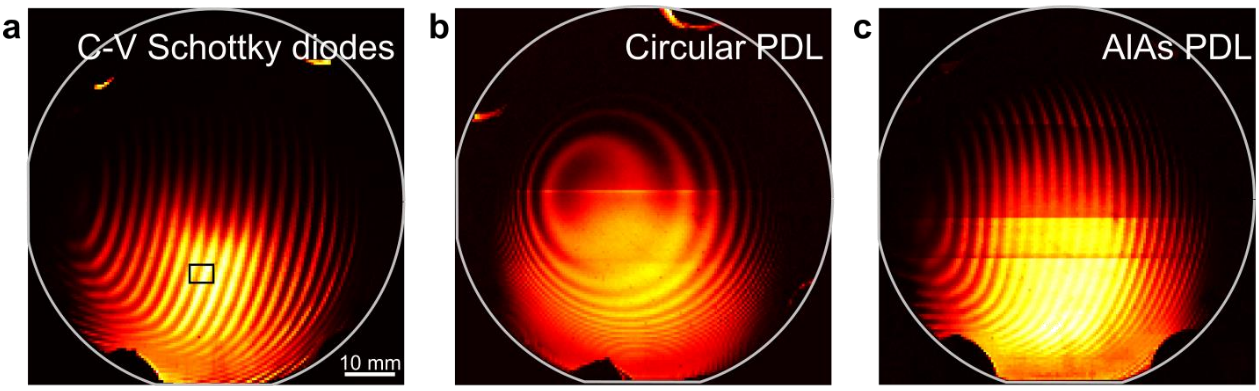

Supplementary Figure 1 Photoluminescence (PL) measurements performed on additional samples.
a Sample wafer used for C-V measurement presented in Supplementary Figure 2. The C-V data presented in Supplementary Figure 2 was recorded from Schottky diode samples processed from the marked region on the wafer. **b** 75 nm thick $Al_{33}Ga_{67}As$ PDL with wafer rotation resulting in a circular pattern. **c** 15 nm thick AlAs PDL. Horizontal line discontinuities in the PL intensity maps originate from interrupted cooling during the line-scan PL map.

QD density measurements using capacitance voltage spectroscopy

To further support that the islands observed in the AFM measurements are the relevant optically active QDs, we performed capacitance-voltage (C-V) spectroscopy measurements. The obtained capacitive signal is directly proportional to the absolute number of QDs below the gate electrode (see below for a description of the samples). In Supplementary Figure 2a we present typical results obtained from devices fabricated at locations on the wafer having the lowest and highest QD densities ρ_{QD}, respectively. We observe two charging peaks of a first and a second electron in each QD of a Schottky diode sample. C-V integrated over the voltage range corresponding to the inhomogeneously broadened charging peak increases as an increasing

number of QDs can be charged. ρ_{QD} values extracted from this are plotted in Supplementary Figure 2b along with the PL intensity at these locations. In agreement with the PL intensity modulation, the obtained local QD densities at this specific area on the wafer range from 4 µm^{-2} to 11 µm^{-2}, similar to the values obtained from uncapped QDs in AFM-measurements (cf. Figure 3).

For C-V measurements, n-i-Schottky diodes were processed from the wafer piece marked with a black outline in Supplementary Figure 1a. n-contacts are formed by indium-solder to the sample corners. The Schottky contacts are 100 nm thick 300 x 300 µm² gold gates, with a 2 nm chrome adhesion layer, that are bonded inside a 16 pin carrier, using ultrasonic wedge bonding. Measurements were performed at liquid Helium temperatures (4.2 K) using an ac voltage at 2333 Hz with 10 mV amplitude (rms) superimposed to a DC voltage (swept to align the band with the QD energy levels) and a lock-in amplifier. The QD density ρ_{QD} extracted from the diode-background subtracted capacitance C of the finite twofold degeneracy of the lowest energy orbital states is given by

$$\rho_{QD} = \frac{\lambda}{2e\ A_G} \int C\ dV$$

where λ is the ratio of the distances between the n-doped back contact and i) the Schottky gate and ii) the QDs, e the elementary charge, V the dc gate voltage and A_G the surface area of the gate[1].

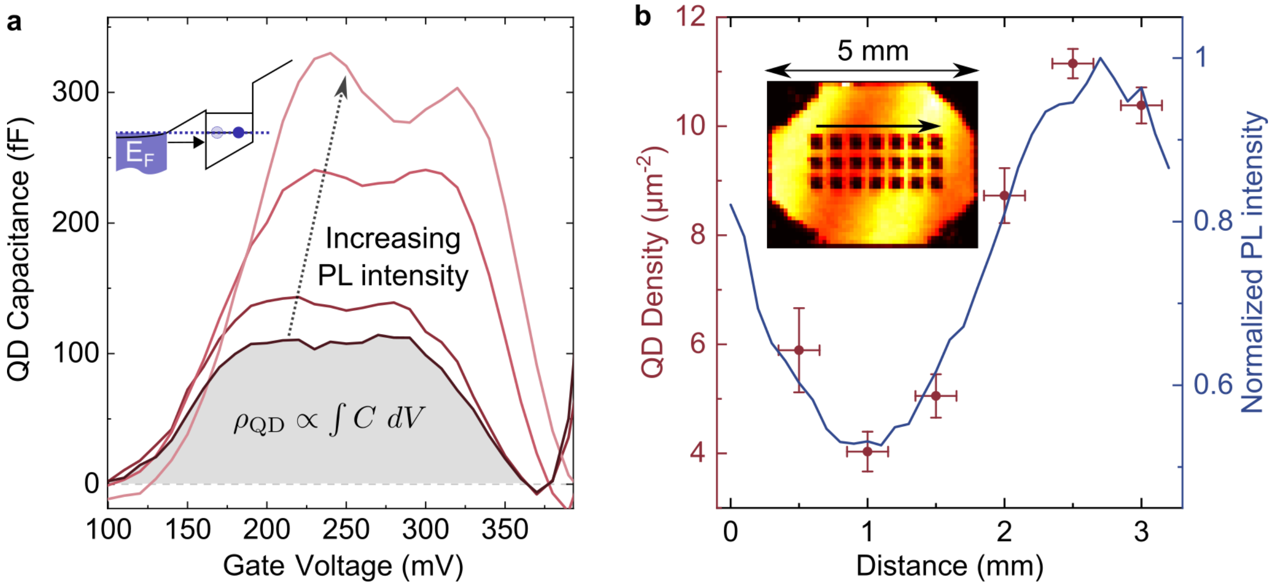

Supplementary Figure 2 Complementary QD density measurements using capacitance voltage spectroscopy. a Diode-background subtracted Capacitance-Voltage spectra of buried QDs along the PL intensity modulation. The inset shows a sketch of the sample bandstructure at a voltage, where electrons can tunnel from the Fermi level of the n-doped back contact E_F to the QD ground state. **b** QD densities deduced by C-V spectroscopy (red dots) and PL intensity (blue line) are plotted versus distance on the sample. The inset shows a PL map of the processed area (same color scheme as in Supplementary Figure 1). Black regions are metallization of the back-contacts in the corners and Schottky-gates, respectively. Error bars in the QD density represent the standard error derived from gaussian fitting of CV data. Error bars in the position represent the width of the top gates.

Detailed study of local contrast

Supplementary Figure 3a shows a PL map of the same sample as used in Figure 1c. In Supplementary Figure 3b, we plot the Michelson-contrast of the PL map by comparing spectrally integrated ranges for high and low intensity by creating polynomial envelope functions:

$$c_{\text{Michelson}} = \frac{I_{\text{high}} - I_{\text{low}}}{I_{\text{high}} + I_{\text{low}}},$$

with I_{high} the upper and I_{low} the lower envelope (cf. Supplementary Figure 4c). In all samples, highest local contrast is found in regions with an InAs amount close to the critical amount Θ_{C}, *i.e.* the onset of QD nucleation.

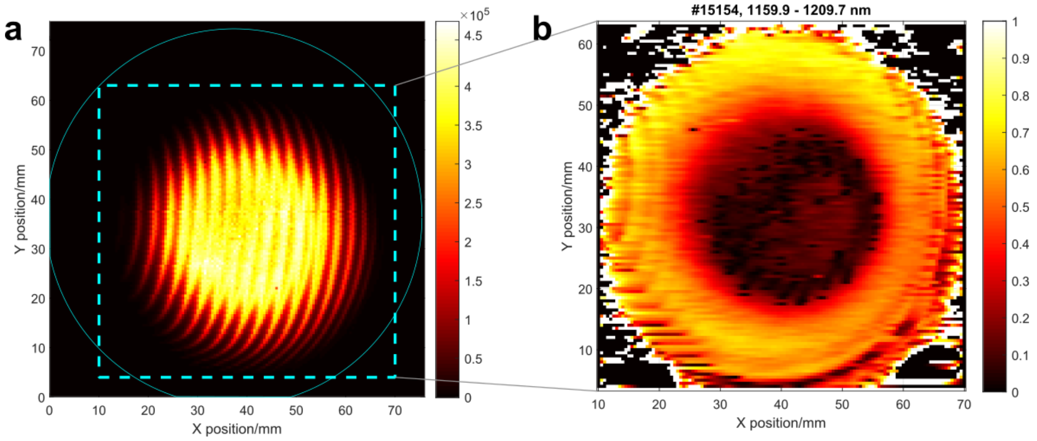

Supplementary Figure 3 Photoluminescence and Michelson-contrast for $t_{\text{anneal}} = 0$ s sample. **a** PL map. **b** Michelson contrast of the marked region in **a** (blue dashed box).

In Supplementary Figure 4 we show a sample exhibiting a high contrast. At contrast values approaching 1 (in the region of 20 – 30 mm), differentiating extremely high contrasts is difficult.

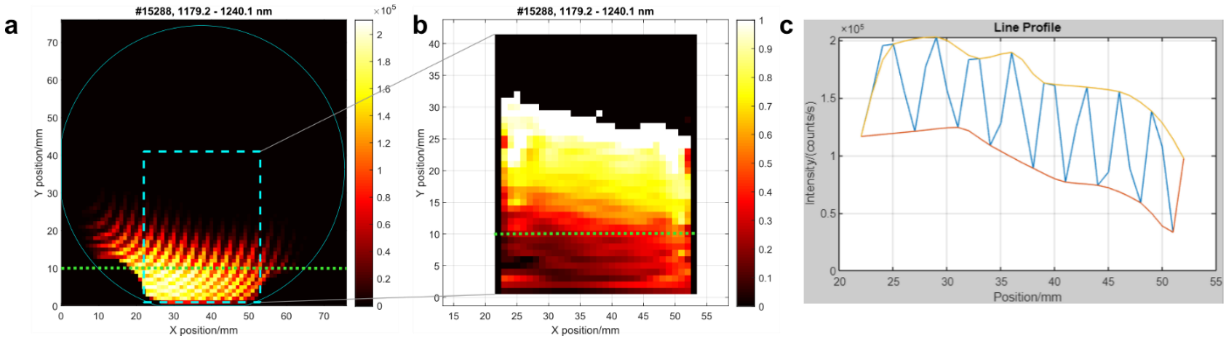

Supplementary Figure 4 Photoluminescence and Michelson-contrast for a high contrast sample. **a** Full wafer PL map. **b** Michelson-contrast of the marked region in **a** (blue dashed box). Note

that the QD nucleation onset region is located at y = 20-30 mm, where due to scaling no luminescence is visible in **a**. **c**, PL intensity (blue line) along the green dotted line from subfigure **a** and upper (yellow) and lower (red) envelope functions.

Supplementary Figure 5 uses the Weber contrast $c_{\text{Weber}} = (I_{\text{high}} - I_{\text{low}}) / I_{\text{low}}$, since it is more suitable for visualizing high contrast values. High Weber-contrasts $c_{\text{Weber}} > 100$ are found, as plotted in Supplementary Figure 5b. Since these high contrasts are found at very low QD densities, the comparison in Figure 1d was made from locations at similar QD densities for the different samples. As a measure for QD density, we used the relation of the s-s and p-p transitions of the QD PL emission. In our setup, locations of equal transition counts correspond to a medium density of ~ 10 QDs/µm^2.

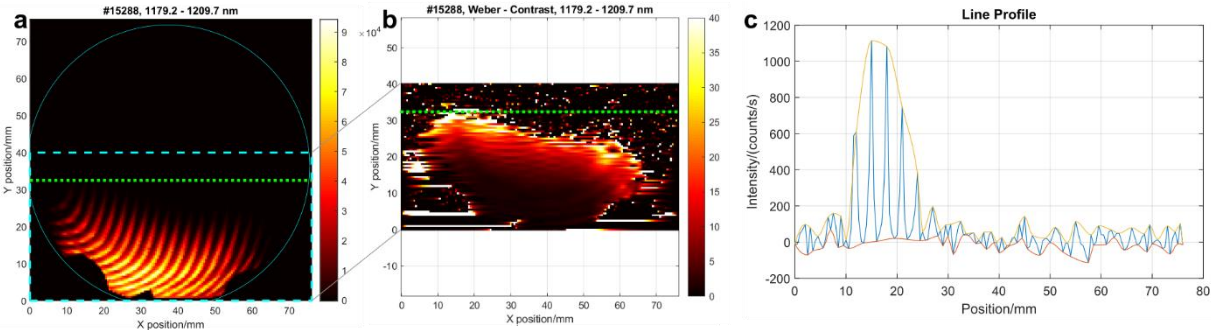

Supplementary Figure 5 Photoluminescence and Weber-contrast for a high contrast sample. **a**, Full wafer PL map. **b** Weber-contrast of the marked region in **a** (blue dashed box). Note that the QD nucleation onset region is located at y = 20-30 mm, where due to scaling no luminescence is visible in **a**. **c** PL intensity (blue line) along the green dotted line (at the onset of QD nucleation) from subfigure **a** and upper (yellow) and lower (red) envelope functions.

Hints of density modulation for local droplet etched quantum dots

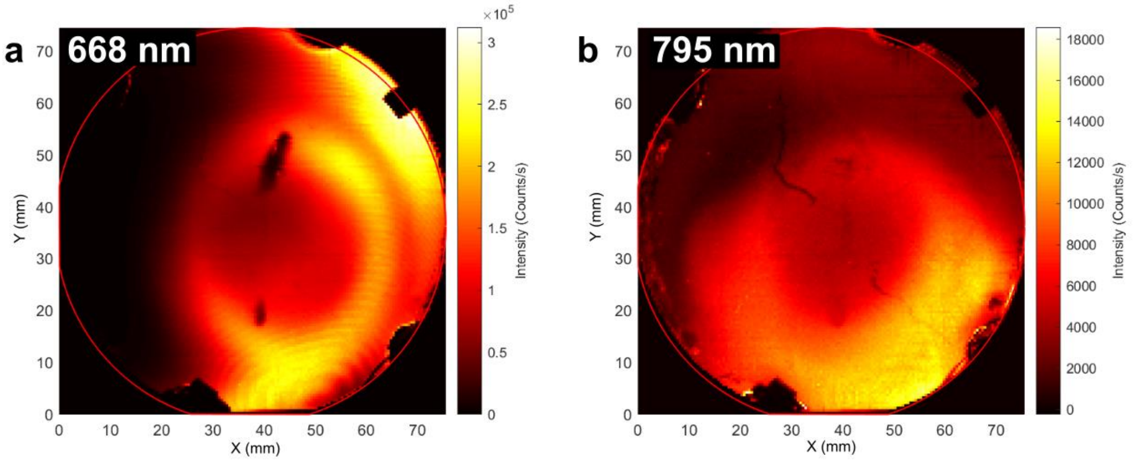

Supplementary Figure 6 Photoluminescence measurements performed on local Al droplet etch QD. a PL map at 668 nm. **b** PL map of the GaAs quantum dots at 795 nm in the same sample.

In Supplementary Figure 6, we present PL-data of local droplet etched QDs. We grow an AlGaAs PDL with a nominal thickness of 30 nm at the centre of the wafer oriented from bottom right to top left. Despite local droplet etching being not a Stranski-Krastanov growth method, we find a faint modulation of the intensity in the PL map. Possible reasons for the weak contrast are a high growth temperature and long annealing breaks necessary for local droplet etch dot epitaxy after the PDL growth[2].

Wafers used for assessing and benchmarking the quality of the QDs

Supplementary Figure 7 presents sample wafer #14843 which consists of a high reflectivity distributed Bragg reflector (DBR) structure with a n-i-p-diode on top. After the n-layer, a tunnel barrier acting as a pattern defining layer was grown under substrate rotation. A PL map of an unprocessed wafer part is presented in Supplementary Figure 7a. Supplemantary Figure 7b shows a simulation of the circular grown PDL. In the simulation the density modulation is based on layer thicknesses determined from quantum well emission. We attribute layers with integer monolayers thicknesses a standard nucleation probability and half monolayer thicknesses an enhanced probability with sinusoidal modulation in between. This simple assumption shows qualitative agreement with the experimental wafer map. As a comparison, in Supplementary Figure 7c, a simulation without a PDL is shown, which would result in an unmodulated distribution of QDs. In Supplementary Figure 7d we show a resonance excitation scan of a neutral exciton transition demonstrating a linewidth of 1.37 μeV which corresponds to ~1.15 times the natural linewidth, determined by lifetime measurements performed on QDs located in an area marked by the red box. By looking at a positively charged trion X^+ in a QD present in the sample under pulsed excitation we measure the second order autocorrelation function and demonstrate anti-bunching with a $g^2(0) = 0.01$, as can be seen in Supplementary Figure 7e. In Supplementary Figure 7f we show the raw indistinguishability measurements by comparing the central peak area at $\tau = 0$ delay for co- and cross-polarized photons and demonstrate a state-of-the-art indistinguishability with a HOM-visibility of V = 0.94. Further measurements of dots from this wafer shown by Najer et al.[3] , as well as Tomm et al.[4] , have demonstrated brightness in the GHz rate regime, an efficiency of more than 50% and coherence over thousands of consecutively emitted photons.

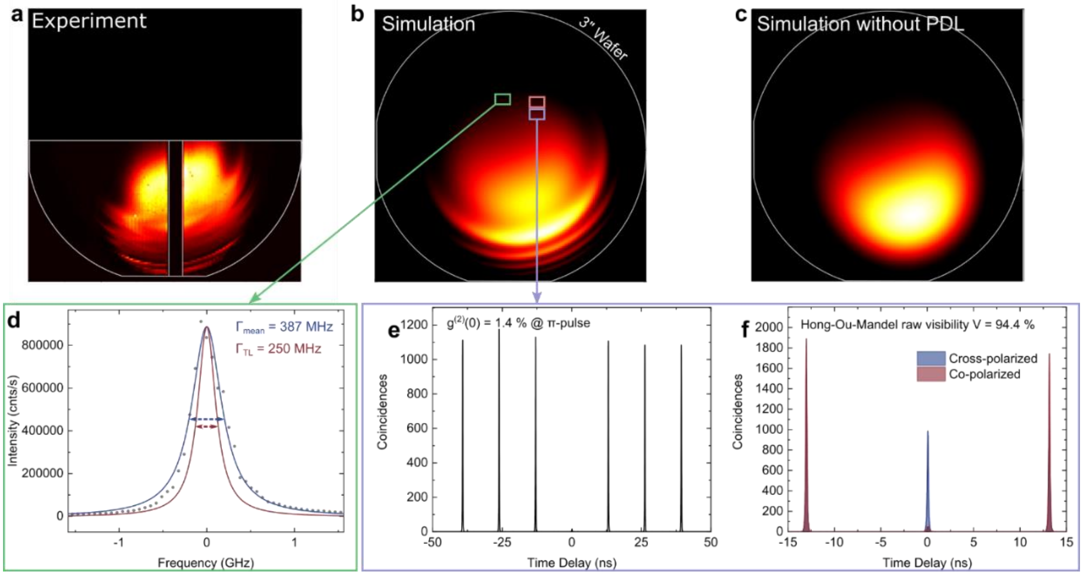

Supplementary Figure 7 Photoluminescence and quantum optics measurements of wafer #14843 with a circular PDL and patterned QD distribution. a PL map of the quantum dots. **b** Simulation of the circular PDL and resulting quantum dot distribution. The green box marks the area for the resonant linewidth scan, the purple box marks the area for anti-bunching and indistinguishability measurements. **c** Simulation of the same layer structure but without PDL. A homogeneous dot distribution is visible. **d** Resonant linewidth scan of a neutral exciton. Blue dots represent the measured values, the blue line is a Lorentz fit of the data. The red line represents the transform limited linewidth. **e** Second-order auto correlation measurement of a positively charged trion X^+ showing a $g^2(0)$ = 0.01. **f** Two-photon correlation measurement showing a HOM-visibility between co- (red area) and cross-polarized photons (blue area) of V = 0.94.

Supplementary Figure 8 presents sample wafer #14813 which also consists of a DBR structure with a n-i-p-diode on top. After the n-layer, a tunnel barrier acting as a pattern defining layer was grown. Samples from this wafer have been processed and used to measure physical quantities, such as the single-electron tunneling rate into a quantum dot, that require an ultra-low noise environment for the dots[5-9].

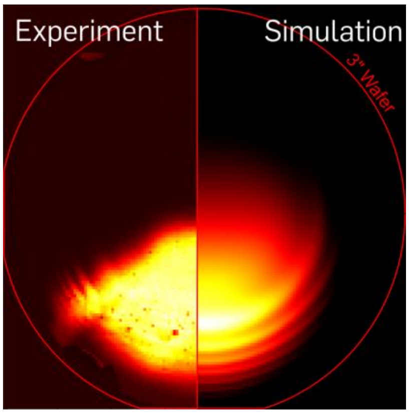

Supplementary Figure 8 Photoluminescence measurements and simulation of Wafer #14813. Devices processed from areas with low PL intensity (and therefore low QD density) have been used for the ultra-low-noise measurements[5-9].

Supplementary Figure 9 presents sample wafer #15027 which consists of QDs embedded in a n-i-p diode. A sacrificial AlGaAs layer below the n-layer allows for fabrication of a thin photonic membrane. The tunnel barrier grown after the n-doped contact layer serves as a circular PDL. In Supplementary Figure 9a we show a PL map that demonstrates the quantum dot density modulation on the wafer. In Supplementary Figure 9b we show a simulation of this area which agrees with the measurement. Supplementary Figure 9c shows a full wafer simulation with colored boxes indicating from which area samples have been processed and measured. In Supplementary Figure 9d we demonstrate the excellent noise-free quality of the dots by showing a resonant linewidth of 568 MHz corresponding to ~1.14 times the natural linewidth. We then verify the state-of-the-art quantum optics properties, as can be seen in Supplementary Figure 9e, f. We achieve an anti-bunching of $g^{(2)}(0) = 0.02$ and a HOM-visibility of V = 0.93. Further measurements using dots from this wafer (Supplementary Figure 9c, purple box) demonstrate the integrability with excellent quantum photonic properties, such as near transform-limited linewidth and a coherent spin-photon interface by Uppu et al.[10,11], Pedersen et al.[12] and Appel et al.[13].

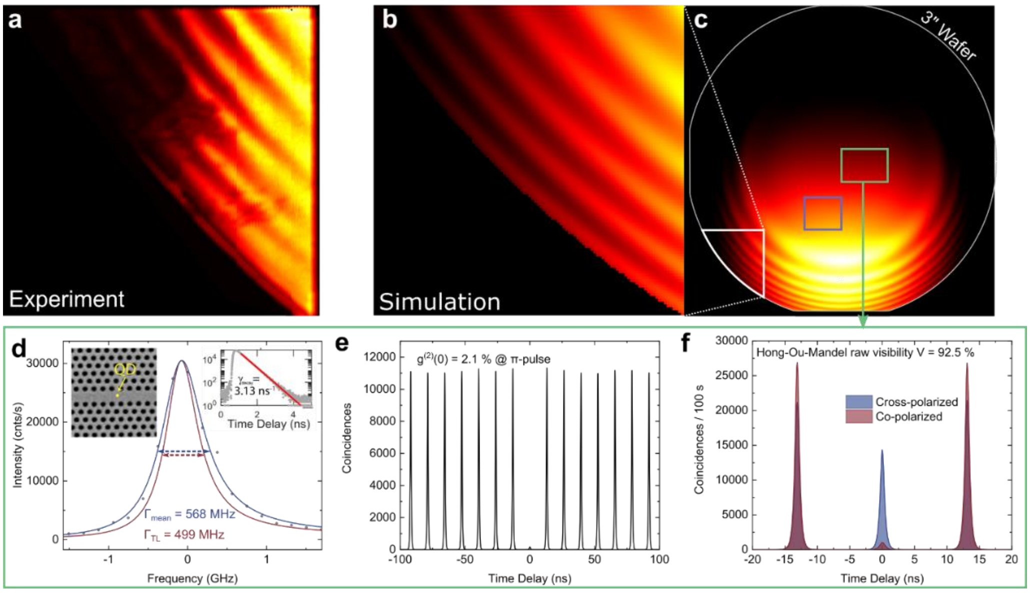

Supplementary Figure 9 Photoluminescence and quantum optics measurements and simulation of Wafer #15027. a PL map of a waferpiece. **b** Simulation zoom-in of the layer structure for the experimentally measured wafer piece. **c** Simulation of the full 3" wafer. The area with a white outline corresponds to the area shown in subfigure b. The green box marks the area for the quantum optics measurements and the purple box marks the area from which further measurements have been performed[10-13]. **d** Resonant linewidth scan showing a linewidth of 568 MHz. The left inset shows an SEM image of the nanophotonic waveguide. The right inset shows lifetime measurements with an exponential fit and the decay rate γ_{decay}. **e** Pulsed second-order autocorrelation showing an anti-bunching of $g^2(0)$ = 0.02. **f** Two-photon interference measurement showing a HOM-visibility of V = 0.93.

Size analysis of quantum dots using atomic force microscopy

Supplementary Figure 10 shows the width of the QDs, measured as full width at half maximum (FWHM) (red circles) and the total height of the QDs (blue circles) and the number of QDs

found in each 2 x 2 µm² area as discussed in Figure 3 in the main text. We observe slightly larger QDs at low QD densities. Between low density QD regions, the dot size seems to plateau. We find QDs tend to be slightly larger at low densities (height = (16 ± 0.4) nm, FWHM = (24.9 ± 0.1) nm) compared to higher densities (height = (15.4 ± 0.1) nm, FWHM = (24.6 ± 0.1) nm).

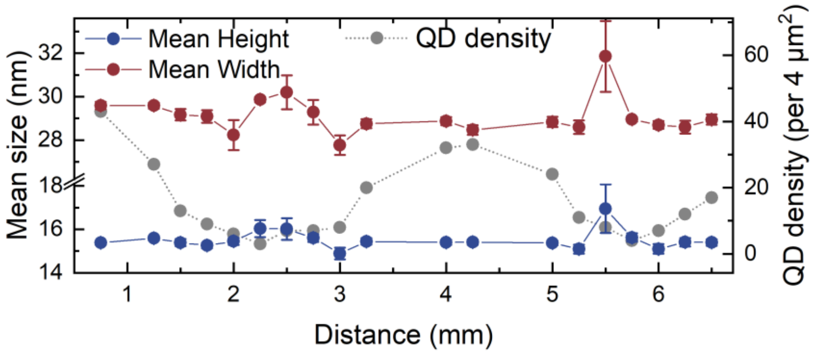

Supplementary Figure 10 Mean size distributions of quantum dots along a PDL. Mean full width at half maximum (blue) and mean absolute height (red) of quantum dots along a density modulation. Error bars indicate the standard error of the mean value. The total number of QDs in a 2 x 2 µm² AFM image is shown in grey. Lines between the points are a guide to the eye.

References

1 Drexler, H., Leonard, D., Hansen, W., Kotthaus, J. P. & Petroff, P. M. Spectroscopy of quantum levels in charge-tunable InGaAs quantum dots. *Phys Rev Lett* **73**, 2252-2255 (1994).

2 Zhai, L. *et al.* Low-noise GaAs quantum dots for quantum photonics. *Nature Communications* **11**, 4745 (2020).

3 Najer, D. *et al.* A gated quantum dot strongly coupled to an optical microcavity. *Nature* **575**, 622-627 (2019).

4 Tomm, N. *et al.* A bright and fast source of coherent single photons. *Nature Nanotechnology* **16**, 399–403 (2021).

5 Lochner, P. *et al.* Contrast of 83% in reflection measurements on a single quantum dot. *Scientific Reports* **9**, 8817 (2019).

6 Kurzmann, A., Ludwig, A., Wieck, A. D., Lorke, A. & Geller, M. Auger Recombination in Self-Assembled Quantum Dots: Quenching and Broadening of the Charged Exciton Transition. *Nano Letters* **16**, 3367-3372 (2016).

7 Kurzmann, A., Ludwig, A., Wieck, A. D., Lorke, A. & Geller, M. Photoelectron generation and capture in the resonance fluorescence of a quantum dot. *Applied Physics Letters* **108**, 263108 (2016).

8 Kurzmann, A. *et al.* Optical detection of single-electron tunneling into a semiconductor quantum dot. *Physical Review Letters* **122**, 247403 (2019).

9 Kurzmann, A. *et al.* Optical blocking of electron tunneling into a single self-assembled quantum dot. *Physical Review Letters* **117**, 017401 (2016).

10 Uppu, R. *et al.* On-chip deterministic operation of quantum dots in dual-mode waveguides for a plug-and-play single-photon source. *Nature Communications* **11**, 3782 (2020).
11 Uppu, R. *et al.* Scalable integrated single-photon source. *Science Advances* **6**, eabc8268 (2020).
12 Pedersen, F. T. *et al.* Near Transform-Limited Quantum Dot Linewidths in a Broadband Photonic Crystal Waveguide. *ACS Photonics* **7**, 2343-2349 (2020).
13 Appel, M. H. *et al.* Coherent Spin-Photon Interface with Waveguide Induced Cycling Transitions. *Physical Review Letters* **126**, 013602 (2021).
