# Supplementary material for: Wafer-scale epitaxial modulation of quantum dot density
Source: Nat Commun. 2022 Mar 28;13:1633. doi: 10.1038/s41467-022-29116-8 (PMC8960873; doi:10.1038/s41467-022-29116-8)
Supplement: Supplementary file 2 — Description of Additional Supplementary Files [file 41467_2022_29116_MOESM2_ESM.pdf]

Inventory of Supporting Information to:

Wafer-Scale Epitaxial Modulation of Quantum Dot Density

‘QD density measurements using capacitance voltage spectroscopy’ is supplemental to Figure 3.

‘Detailed study of local contrast’ is supplemental to Figure 1.

‘Hints of density modulation for local droplet etched quantum dots’ is supplemental to Figure 4.

‘Wafers used for assessing and benchmarking the quality of the QDs’ is related to and mentioned in the discussion section.

‘Size analysis of quantum dots using atomic force microscopy’ is supplemental to Figure 3.

Supplementary Information References

Supplementary Table 1 is related to the method section.

Supplementary Figure 1 is related to Figure 4 and mentioned in the discussion section.

Supplementary Figure 2 is related to Figure 3.

Supplementary Figure 3 is related to Figure 1.

Supplementary Figure 4 is related to Figure 1.

Supplementary Figure 5 is related to Figure 1.

Supplementary Figure 6 is related to Figure 4.

Supplementary Figure 7 is related to and mentioned in the discussion section.

Supplementary Figure 8 is related to and mentioned in the discussion section.

Supplementary Figure 9 is related to and mentioned in the discussion section.

Supplementary Figure 10 is related to Figure 3.
